# S139. INVESTIGATING THE GENETIC ARCHITECTURE OF GENERAL AND SPECIFIC PSYCHOPATHOLOGY IN ADOLESCENCE USING SCHIZOPHRENIA POLYGENIC SCORES

**DOI:** 10.1093/schbul/sby018.926

**Published:** 2018-04-01

**Authors:** Hannah Jones, Jon Heron, Gemma Hammerton, Jan Stochl, Peter B Jones, Mary Cannon, George Davey Smith, Peter Holmans, Glyn Lewis, David EJ Linden, Michael C O’Donovan, Michael J Owen, James Walters, Stanley Zammit

**Affiliations:** 1 University of Bristol; 2 University of Cambridge; 3 Royal College of Surgeons in Ireland; 4 Cardiff University; 5 University College London; 6 University of Bristol, Cardiff University

## Abstract

**Background:**

Whilst associations between polygenic risk scores (PRSs) for schizophrenia and various phenotypic outcomes have been reported, an understanding of developmental pathways can only be gained by modelling comorbidity across psychopathology, something no studies have done to date. We examine how genetic risk for schizophrenia relates to a broad range of adolescent psychopathology using a latent modelling approach, and compare this to genetic risk for other psychiatric disorders, to gain a more comprehensive understanding of development pathways at this age.

**Methods:**

PRSs for schizophrenia, major depressive disorder, neuroticism and bipolar disorder were generated for individuals in the Avon Longitudinal Study of Parents and Children (ALSPAC) birth cohort. Multivariate linear regression was used to examine relationships of these PRSs with psychopathology factors modelled within i) a correlated factors structure, and ii) a bifactor structure.

**Results:**

The schizophrenia PRS was associated with an increase in factors describing psychotic experiences, negative dimension, depression, and anxiety, but once modelling a general psychopathology factor specific effects above this persisted only for the negative dimension. Similar factor relationships were observed for the neuroticism PRS, with a (weak) specific effect only for anxiety once modelling general psychopathology.

**Discussion:**

Psychopathology during adolescence can be described by a general psychopathology construct that captures common variance as well as by specific constructs capturing remaining non-shared variance. Schizophrenia risk genetic variants identified through genome-wide association studies mainly index negative rather than positive symptom psychopathology during adolescence. This has potentially important implications both for research and risk prediction in high-risk samples.

